# Clinical validation of an artificial intelligence algorithm for classifying tuberculosis and pulmonary findings in chest radiographs

**DOI:** 10.3389/frai.2025.1512910

**Published:** 2025-02-06

**Authors:** Thiago Fellipe Ortiz de Camargo, Guilherme Alberto Sousa Ribeiro, Maria Carolina Bueno da Silva, Luan Oliveira da Silva, Pedro Paulo Teixeira e Silva Torres, Denise do Socorro da Silva Rodrigues, Mayler Olombrada Nunes de Santos, William Salibe Filho, Marcela Emer Egypto Rosa, Magdala de Araujo Novaes, Thiago Augusto Massarutto, Osvaldo Landi Junior, Elaine Yanata, Marcio Rodrigues da Cunha Reis, Gilberto Szarf, Pedro Vieira Santana Netto, Joselisa Peres Queiroz de Paiva

**Affiliations:** ^1^Image Research Center, Hospital Israelita Albert Einstein, São Paulo, Brazil; ^2^Electrical, Mechanical and Computer Engineering School, Federal University of Goias, Goias, Brazil; ^3^Infectology Division, Clemente Ferreira Institute, São Paulo, Brazil; ^4^Aparecida of Goiania Municipal Hospital, Hospital Israelita Albert Einstein, Goias, Brazil; ^5^Pulmonary Division, Heart Institute, São Paulo, Brazil; ^6^Medical Sciences Center, Federal University of Pernambuco, Pernambuco, Brazil; ^7^Diagnostic Imaging Research and Study Institute Foundation, São Paulo, Brazil; ^8^Studies and Researches in Science and Technology Group, Federal Institute of Goias, Goias, Brazil

**Keywords:** chest X-rays, artificial intelligence, deep learning, clinical validation, convolutional neural network

## Abstract

**Background:**

Chest X-ray (CXR) interpretation is critical in diagnosing various lung diseases. However, physicians, not specialists, are often the first ones to read them, frequently facing challenges in accurate interpretation. Artificial Intelligence (AI) algorithms could be of great help, but using real-world data is crucial to ensure their effectiveness in diverse healthcare settings. This study evaluates a deep learning algorithm designed for CXR interpretation, focusing on its utility for non-specialists in thoracic radiology physicians.

**Purpose:**

To assess the performance of a Convolutional Neural Networks (CNNs)-based AI algorithm in interpreting CXRs and compare it with a team of physicians, including thoracic radiologists, who served as the gold-standard.

**Methods:**

A retrospective study from January 2021 to July 2023 evaluated an algorithm with three independent models for Lung Abnormality, Radiological Findings, and Tuberculosis. The algorithm's performance was measured using accuracy, sensitivity, and specificity. Two groups of physicians validated the model: one with varying specialties and experience levels in interpreting chest radiographs (Group A) and another of board-certified thoracic radiologists (Group B). The study also assessed the agreement between the two groups on the algorithm's heatmap and its influence on their decisions.

**Results:**

In the internal validation, the Lung Abnormality and Tuberculosis models achieved an AUC of 0.94, while the Radiological Findings model yielded a mean AUC of 0.84. During the external validation, utilizing the ground truth generated by board-certified thoracic radiologists, the algorithm achieved better sensitivity in 6 out of 11 classes than physicians with varying experience levels. Furthermore, Group A physicians demonstrated higher agreement with the algorithm in identifying markings in specific lung regions than Group B (37.56% Group A vs. 21.75% Group B). Additionally, physicians declared that the algorithm did not influence their decisions in 93% of the cases.

**Conclusion:**

This retrospective clinical validation study assesses an AI algorithm's effectiveness in interpreting Chest X-rays (CXR). The results show the algorithm's performance is comparable to Group A physicians, using gold-standard analysis (Group B) as the reference. Notably, both Groups reported minimal influence of the algorithm on their decisions in most cases.

## 1 Introduction

Chest X-rays (CXR) are critical in screening and monitoring respiratory diseases. However, their interpretation can be challenging due to factors such as overlapping anatomical structures (e.g., ribs, clavicles, thoracic spine, pulmonary vessels, heart, mediastinum, and diaphragm), difficulties in visual search and lesion recognition, frequent interruptions, observer inexperience, and poor image quality. These challenges contribute to reading errors, particularly among non-specialists in thoracic imaging (Tack and Howarth, 2019; Del Ciello et al., 2017). In response to these challenges, there has been increasing interest in using artificial intelligence (AI) tools for medical imaging, particularly convolutional neural networks and deep learning (Khan et al., 2021). Significant advancements have been made in developing algorithms to detect pulmonary abnormalities, offering promising solutions to support diagnostic decision-making (Chartrand et al., 2017; Kohli et al., 2017). For example, CXR-based automated systems have been proposed to rapidly detect pneumonia and characterize incidental pulmonary nodules (Mathew et al., 2020).

As with any medical device or health technology, proper validation of AI algorithms is essential for their adoption in clinical practice. This ensures patient safety, maximizes benefits, and mitigates risks of inadvertent harm (de Hond et al., 2022). Validation requires evaluating an algorithm's diagnostic performance across a spectrum of disease manifestations and demographic variations, ensuring bias is minimized (Park and Han, 2018; Park and Kressel, 2018). Thus, prior to clinical adoption, AI algorithms must undergo rigorous validation processes that include internal and clinical validation. Internal validation assesses an algorithm's reliability and generalization in controlled conditions. In contrast, clinical (or external) validation evaluates its efficacy in real-world settings, spanning diverse populations, environments, and imaging equipment (Altman and Royston, 2000). Clinical validation must also reflect the target population, analyzing performance across subgroups defined by age, ethnicity, sex, socioeconomic status, and geographic location (Kelly et al., 2019). This two-phased approach enhances confidence in AI-supported diagnostics, transitioning them from experimental applications to patient-centered practices (Vasey et al., 2022).

Several studies exemplify successful clinical validation efforts. For instance, Hosny et al. (2022) validated a deep learning model for segmenting tumors and lymph nodes in CT images of non-small cell lung cancer, optimizing radiotherapy planning. Ueda et al. (2021) demonstrated the effectiveness of AI-assisted software in improving lung cancer detection in chest X-rays, particularly enhancing general practitioners' accuracy. Similarly, Cid et al. (2024) validated open-source neural networks using extensive datasets from UK hospitals, showcasing robust generalization. Blake et al. (2023) conducted external validation of a CE-marked AI tool, qXR, for stratifying CXRs as normal or abnormal, confirming its high sensitivity and highlighting its potential to reduce reporting delays. Nam et al. (2023) underscored AI's efficacy in lung nodule detection through a randomized clinical trial, while Nguyen et al. (2022) and Thian et al. (2021) emphasized the importance of external validation in deploying AI systems for real-world use.

Despite significant advancements in artificial intelligence, a critical gap persists in validating AI algorithms for tuberculosis (TB) detection in conjunction with other radiological findings, particularly in scenarios involving physicians with varying levels of expertise. While many AI systems are designed to identify multiple conditions on chest radiographs, TB is often under-represented or not prioritized as a primary outcome. Furthermore, in real-world clinical settings, chest radiographs are frequently interpreted by non-specialist physicians or practitioners from other specialties due to the limited availability of board-certified radiologists. This gap-insufficient focus on TB and the reliance on non-specialist evaluations–highlights the urgent need for innovative solutions. Addressing this gap, the present study develops and validates an AI algorithm tailored to classify TB and various radiological findings, leveraging input from physicians with diverse expertise in chest radiograph interpretation. This approach underscores the importance of integrating AI into broader clinical workflows, making this work timely and essential.

This study addresses the gap described above by incorporating physicians' expertise, from non-specialists to radiologists, pulmonologists, and infectious disease experts, to validate an artificial intelligence algorithm designed to analyze chest radiographs, including TB and other radiological findings. The algorithm detects pulmonary abnormality, tuberculosis, and other radiological findings, providing a comprehensive evaluation across diverse clinical perspectives. Its inclusive design addresses the reliance on non-specialist physicians due to a shortage of radiologists, enhancing real-world applicability. By fostering interdisciplinary collaboration, this study strengthens algorithm validation. It highlights the effective integration of AI into patient care, paving the way for robust, inclusive strategies in diagnosing and managing complex diseases like tuberculosis.

Based on the information presented, the following hypothesis is formulated: if efficient external validation processes have been applied to artificial intelligence algorithms for the analysis of chest radiographs, then it is feasible to apply the external validation technique for the identification of pulmonary abnormality, tuberculosis, and nine other radiological findings with the support of physicians from various specialties. This process will increase the robustness and clinical applicability of artificial intelligence algorithms and ensure that the algorithm can be effectively used in a real clinical environment, where the diversity of interpretations and the complexity of cases reflect the multidisciplinary nature of medical diagnosis.

Few studies have clinically validated AI algorithms in medical imaging using external data that accurately reflect real-world scenarios, particularly involving physicians from various specialties (Kim et al., 2019). Therefore, the main objective of this work is to develop and clinically validate an AI algorithm comprising three models to classify: (i) Tuberculosis, (ii) Radiological Findings, and (iii) Pulmonary Abnormality from chest radiographs. By initiating an innovative approach to clinical validation, this work seeks to assist in interpreting CXRs and serve as a guide for researchers to enhance AI algorithms for broader clinical applications. The specific objectives are (i) datasets' separation, (ii) models' construction, (iii) training and internal validation, and (iv) retrospective clinical validation of the proposed models.

The paper is structured as follows: (i) Section 2 presents the methodology used to develop algorithms and clinical validation, (ii) Section 3 brings the results and some analyses, and (iv) Section 4 presents the conclusions and future direction about the clinical validation of AI algorithms with physicians of different specialties.

## 2 Materials and methods

This section presents the main methodological aspects of this study and some theoretical aspects (Artificial Intelligence, Model Development, and Clinical Validation) to serve as a basis for understanding the methodology of this work.

### 2.1 Artificial intelligence model development

Convolutional Neural Networks (CNNs) were utilized to develop the algorithm, which consists of three models that work independently of each other: (i) the Lung Abnormality Model (LAM) to classify the lung parenchyma image as normal or abnormal, (ii) the Radiological Findings Model (RFM) to classify specific findings on CXR into nine classes (consolidation, lung opacity, lung injury, atelectasis, edema, pneumothorax, pleural effusion, cardiomegaly, and mediastinal widening), and (iii) Tuberculosis Model (TBM) to determine whether the CXR is compatible with pulmonary tuberculosis or not.

The architecture of these models consists of a pre-trained backbone (DenseNet-121 for LAM and TBM, and DenseNet-169 for RFM, with ImageNet weights) and a dense convolution layer for classification, with weights initialized using the normal distribution (Deng et al., 2009). Weakly supervised learning associated with the Probability Class Activation Map is applied to strengthen the models' ability to localize the radiological findings (Zhou, 2018; Ye et al., 2020). The input images were resized to a 512 × 512 matrix size. When resizing was necessary, zero-filling was used in conjunction with linear interpolation to maintain the image's original aspect ratio. The exclusion criteria employed during the model training phase were the same as those approved by the ethics committee for the external validation phase. This uniformity ensures that the entire study complies with the established ethical guidelines, guaranteeing the integrity and reliability of the results obtained.

Regarding hyperparameters, the optimizer used was Lookahead Adam, and the loss function was binary cross-entropy. The Tuberculosis and Radiological Findings models were trained for 30 epochs with an initial learning rate of 0.0005 and 0.001, respectively. The batch size of the TBM was 16 and of the RFM was 32. The Lung Abnormality model, on the other hand, was trained for 15 epochs with an initial learning rate of 0.0005 and a batch size of 16. Additionally, a learning rate scheduler was employed: for the TBM, it was multiplied by 0.05 every 5 epochs; for the LAM, it was multiplied by 0.8 every 3 epochs; and for the RFM, it was multiplied by 0.95 every 2 epochs. The learning rate and the number of epochs were empirically adjusted to optimize performance and avoid overfitting. To address the class imbalance in the dataset, loss weights were applied (Japkowicz and Stephen, 2002).

Each model's training, validation, and test subsets were randomly selected at the patient level to prevent data leakage. For each of the three models, the training subset consisted of 70% of the original dataset, while the remaining 30% was allocated to the internal validation subset (20% for validation and 10% for test). The area under the receiver operating characteristic curve (AUC) metric was calculated on the validation and test subsets to evaluate the performance of the three models. Each algorithm provide a classification score and generates a heatmap to enhance the explainability of the results.

PyTorch 1.5.0 was used for algorithm development in Python (version 3.6, Python Software Foundation) (Paszke et al., 2019).

#### 2.1.1 Datasets used for the AI development

The development of the algorithm employed a total of 252,721 selected images from the CheXpert, Tuberculosis Portals, PadChest, NIH ChestX-ray8, Montgomery, and Shenzhen datasets (Irvin et al., 2019; Rosenthal et al., 2017; Bustos et al., 2020; Wang et al., 2017; Jaeger et al., 2014). Table 1 shows a summary of class distribution, original image resolution, and post-processing techniques. To ensure consistency, the patient exclusion criteria used during the model training phase were the same as those approved by the ethics committee for the external validation phase. Patients over 18 years old were excluded, as were images acquired with projections other than frontal. Additionally, images of unacceptable quality–such as those not encompassing the entire thorax or those overexposed to X-rays, which limit diagnostic capability–were excluded.

**Table 1:** Summary of class distribution, original resolution, and post-processing techniques for the datasets used.

	**Class distribution**	**Original resolution**	**Post-processing steps**
ChestX-ray8	Atelectasis: 11,559 Cardiomegaly: 2,776 Pneumothorax: 5,329 Lung opacity: 19,990 Lung lesion: 6,326 Pleural effusion: 13,649 Others: 49,319	2,000 × 3,000 pixels	Not mentioned
CheXpert	Atelectasis: 29,333 Cardiomegaly: 23,002 Mediastinal widening: 9,020 Pneumothorax: 17,313 Lung opacity: 92,669 Lung lesion: 6,856 Pleural effusion: 75,696 Others: 34,427	372 × 325 (mean)	Automatic labels with NLP rules
PadChest	Tuberculosis: 308 Tuberculosis sequelae: 1,070 Cavitation: 620 Calcified adenopathy: 647 Granuloma: 732 Calcified granuloma: 3,198 Apical pleural thickening: 3,625 Other: 319,917	High resolution, not dimensioned	Pre-processing with DICOM window
Montgomery (MC)	TB Positive: 58 TB Negative: 80	4,020 × 4,892 and 4,892 × 4,020	Manually segmented masks
Shenzhen	TB Positive: 336 TB Negative: 326	Approximately 3,000 × 3,000 pixels	Not mentioned
TB Portals	TB Positive: 1,129 TB Negative: 170	2,568 × 2,352 (mean)	Not mentioned

The original database labels were used, with the exception of the PadChest dataset for the TB model, in which the following labels were considered positive for tuberculosis: (i) tuberculosis, (ii) sequelae tuberculosis, (iii) cavitation, (iv) calcified adenopathy, (v) granuloma, (vi) calcified granuloma, and (vii) apical pleural thickening.

For the Lung Abnormality model, the PadChest and the Tuberculosis Portals datasets were not used, resulting in a total of 51,879 images from 30,204 patients (male-to-female ratio 1,916/6,593; mean age 52.88 years ± 18.77 [standard deviation (SD)]. The ratio of positive to negative cases was 25,879/26,000, respectively.

For the Radiological Findings model, only the multilabel CheXpert dataset was used, comprising a total of 191,211 images from 64,540 patients (male-to-female ratio 13,953/30,259; mean age 60.67 years ± 17.82 SD). The number of positive cases for each class was as follows: consolidation (16,900), atelectasis (29,793), lung opacity (111,707), lung lesion (7,041), edema (49,716), pneumothorax (17,700), pleural effusion (76,958), cardiomegaly (23,450), mediastinal widening (7,391), and 30,007 images showed no findings. It is pertinent to note that the possibility of encountering multiple findings within a single image exists, highlighting the complexity of radiological diagnosis and the comprehensive nature of the CheXpert dataset in capturing such diversity.

All datasets except NIH ChestX-ray8 were used for the TBM, resulting in a dataset of 2,554 images from 2,292 patients (male-to-female ratio 82/207; mean age 59.56 years ± 17.48 SD). The ratio of positive to negative cases was 67/605, respectively.

All images underwent preprocessing steps including histogram equalization, Gaussian blur, and normalization. Regarding some ethical aspects and patient privacy for datasets used during model training, validation, and testing, all images from these public datasets are anonymized from their origin. Some datasets share information related to gender and age (for example, CheXpert), but not all of them.

### 2.2 Validation study design

Clinical validation involves rigorously testing a new medical technology or method against established clinical benchmarks and standards. In the case of AI algorithms in radiology, this would mean comparing the algorithm's diagnostic accuracy, reliability, and safety against current best practices or gold standards in radiology (Briganti and Le Moine, 2020). The process often includes statistical analysis of the algorithm's performance in various clinical scenarios, assessment of its usability in real-world settings, and evaluation of its impact on patient outcomes (Park, 2019).

Recent advances have shown that AI algorithms can help radiologists improve their performance in detecting certain diseases in radiologic images (Li et al., 2021). However, challenges such as algorithm bias, data privacy, and integration into clinical workflows still need to be addressed (Saw and Ng, 2022).

The validation study was managed as a retrospective study. The retrospective validation stage was conducted with the objective of comparing the performance of physicians with varying levels of experience in interpreting chest radiographs with that of the algorithms. Among those physicians, there were pulmonologists, infectious disease specialists, neurologists, and radiology residents. These physicians were referred to as Group A (GA) physicians for ease of writing and understanding. This group represents many real-world settings because chest radiographs are often interpreted by non-specialist physicians or physicians with other specialties due to the lack of board-certified radiologists. Therefore, involving Group A mirrors the actual clinical environments where the AI algorithm is intended to be used to support the clinical decision.

Radiologists specialized in thoracic image interpretation were designated as the gold standard. These physicians are responsible for generating the ground truths of the images evaluated in the retrospective phase. They were referred to as Group B (GB) physicians.

**Figure 1:**
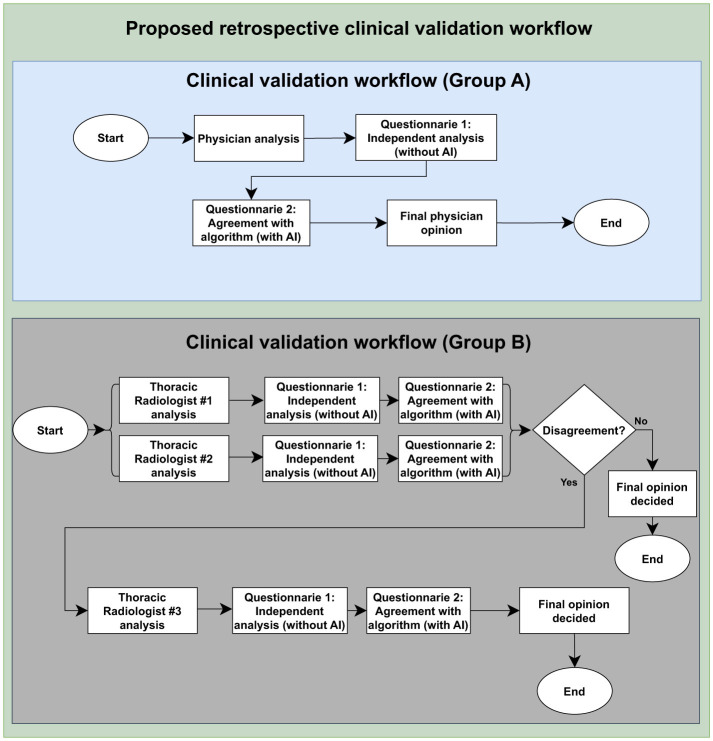
Clinical validation study design. The flow highlights the assessment of agreement between the algorithm results and the evaluations conducted by Group A and board-certified thoracic radiologists, Group B, as well as the Group A personal analysis collection and the ground truth construction by Group B. To access the questionnaire, please see Supplementary material.

The Group A team individually analyzed a set of CXR and classified the exams as “normal” or “abnormal.” When the image was abnormal, they classified the radiographic alterations into nine classes: consolidation, lung opacity, lung injury, atelectasis, edema, pneumothorax, pleural effusion, cardiomegaly, and mediastinal widening. The evaluator also predicted the chance of the image being compatible with tuberculosis. Lastly, the scientists signaled the abnormality's position using an anatomical approach shown in Figure 1. At this point, neither clinical nor laboratory data were available.

Following their own evaluations, physicians could consult the results of three AI models that supported the chest x-ray interpretation. This assessment was conducted using a questionnaire administered through the Open Health Image Foundation (OHIF) visualization platform (Ziegler et al., 2020) and Google Forms. To avoid influence bias, physicians were trained to consult the AI's decision only after doing their personal readings.

**Figure 2:**
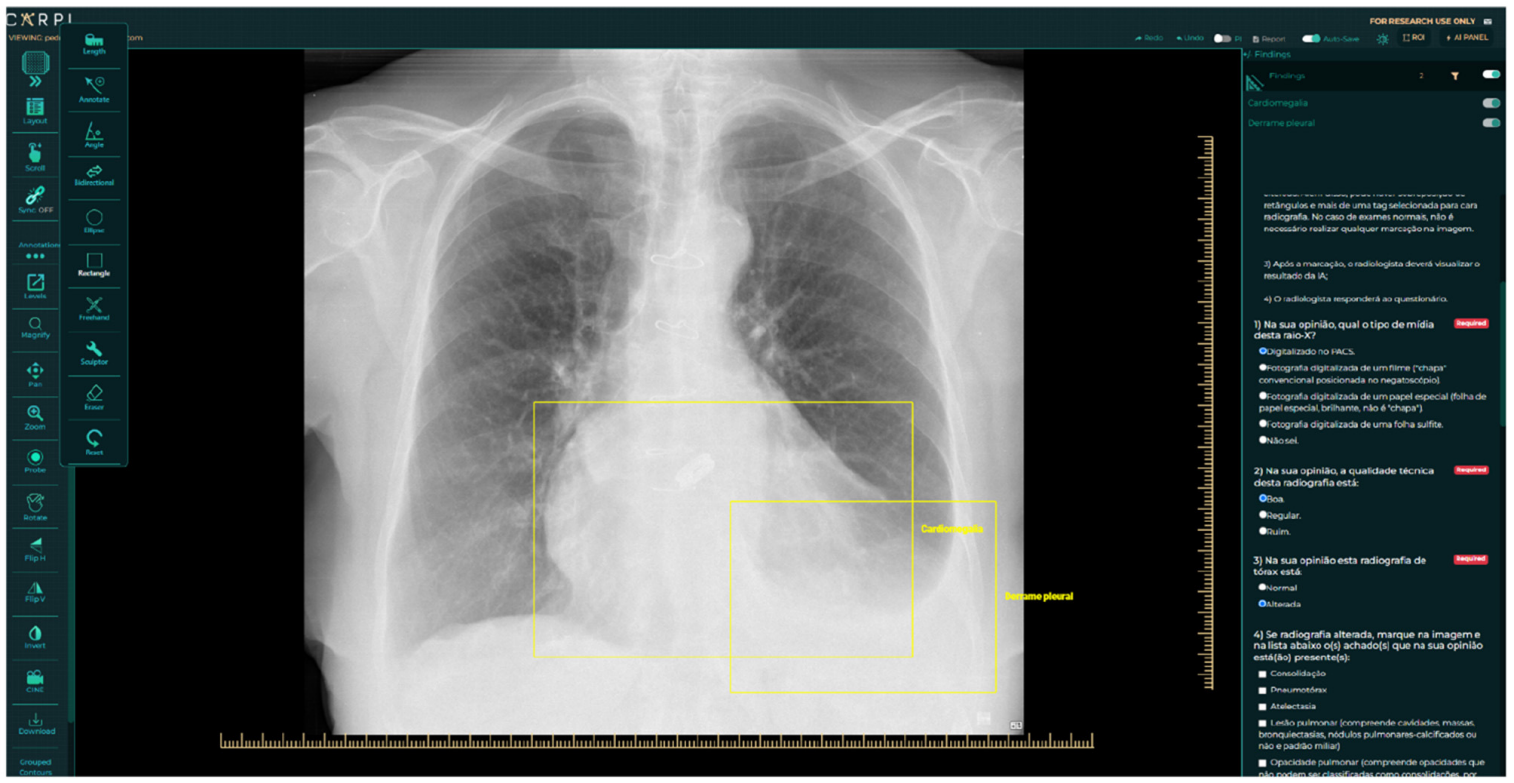
Annotation and questionnaire presentation example on CARPL Platform. Bounding boxes delineate Pleural Effusion and Cardiomegaly.

The next step involved assigning two radiologists with specialized training in cardiothoracic radiology to interpret every image (also detailed in Figure 1). They classified each CXR as “normal” or “abnormal,” categorized the radiographic findings into those nine patterns, and determined whether the image was compatible with tuberculosis. A third, more senior radiologist ( ≤ 5 years of experience), was consulted to break any ties in the analyses if there was any disagreement between the two experts. In addition to classifying the images, the experts manually marked them with bounding boxes to identify where changes occurred in the CXR. The platform used for this task was the CARPL Platform, as illustrated in Figure 2 (CARPL.AI PVT LTD., Delhi, India).

As demonstrated, radiologists specializing in cardiothoracic imaging served as Group B. The images used in this retrospective phase were provided from public databases available on the web [BRAX (Reis, 2022), PadChest (Bustos et al., 2020), and Tuberculosis Portals (Rosenthal et al., 2017) datasets]. The exclusion criteria cited in Section 2.1.1 was the same used in this validation process. Regarding some ethical aspects and patient privacy for datasets used during the validation process, all images from these public datasets are anonymized from their origin. Moreover, it was ensured that a proportional standard was maintained in the number of cases relative to each finding. This methodological choice aimed at preserving the integrity and the representativeness of the sample, guaranteeing that the analysis mirrors the real-world distribution of thoracic conditions. The idea of this validation is to check (in general) if the AI algorithm can help physicians who are non-specialists in chest radiograph interpretation to make good clinical decisions together.

### 2.3 Statistical analysis

The statistical analysis of the retrospective study consisted of two main analyses. Firstly, the classification performance of Group A and the algorithm was compared. This comparison involved assessing accuracy, sensitivity, specificity, and positive and negative predictive values (PPV and NPV, respectively). All metrics were calculated using the Group B analysis as the reference standard, and the Group A image classification results were collected through questionnaire responses. Confidence intervals for specificity, sensitivity, and accuracy were calculated using the Clopper-Pearson methodology (Clopper and Pearson, 1934), while confidence intervals for PPV and NPV were computed using the Mercaldo's Logit Method (Mercaldo et al., 2007).

Secondly, this study utilized descriptive statistics to assess the agreement between Group A and Group B concerning the algorithm's heatmap. Physicians were asked to indicate their level of agreement, whether they agreed, partially agreed, or disagreed with the heatmap's findings. Additionally, the impact of the algorithm on physicians' perspectives was evaluated, with participants being asked if the algorithm had influenced their diagnostic opinions.

## 3 Results

This section aims to present the main results of the article, from the model's internal validation process to the retrospective validation process.

### 3.1 Artificial intelligence model development results

**Figure 3:**
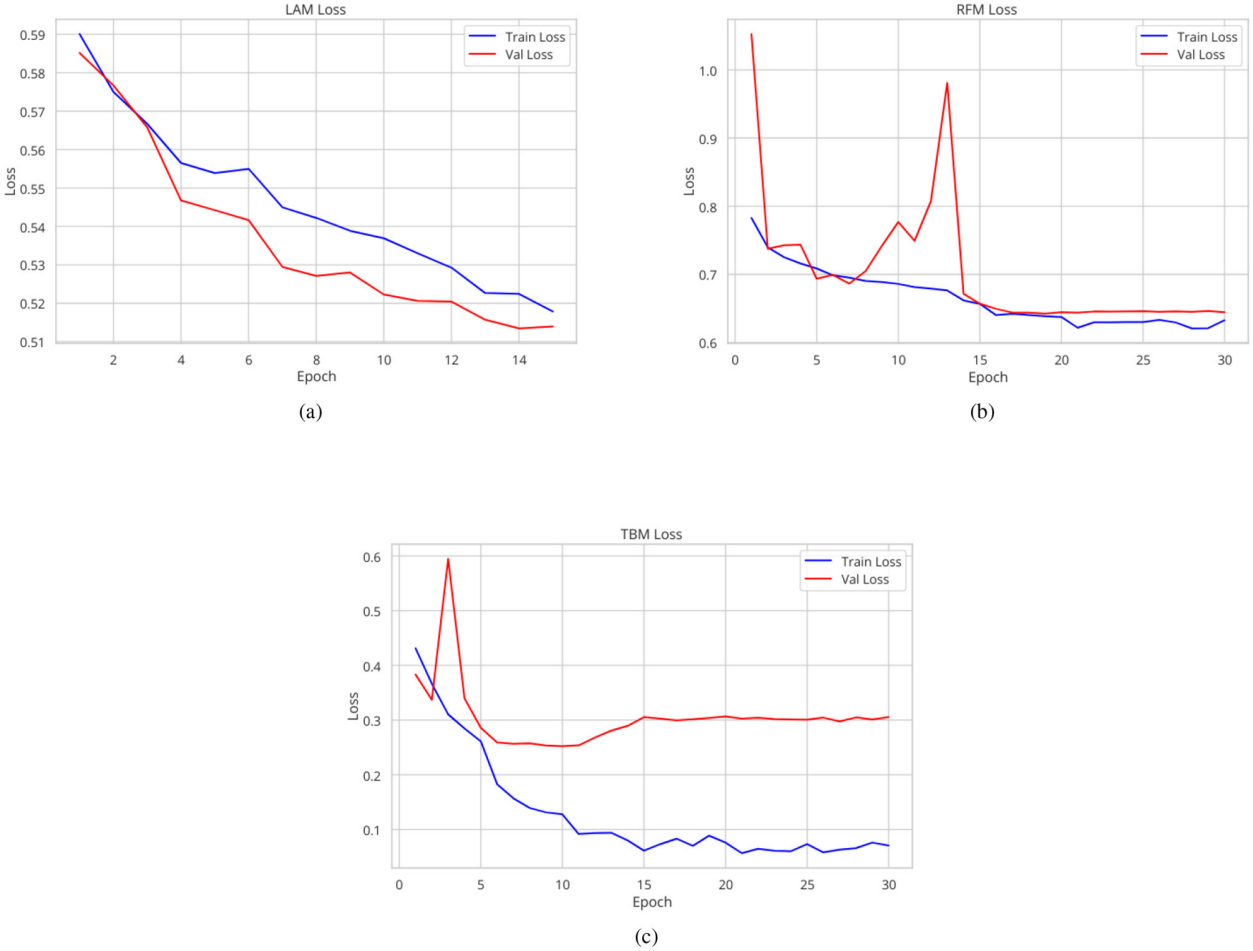
Training and validation loss curves for the LAM **(A)**, the RFM **(B)**, and the TBM **(C)** models.

The Lung Abnormality model (LAM), a binary model (0: normal; 1: abnormal), tested on the CheXPert, NIH, Montgomery, and Shenzhen datasets using a different subset from the training set, achieved an AUC of 0.94. The Tuberculosis model (TBM), also a binary model (0: normal for TB; 1: abnormal for TB), tested on the Tuberculosis Portals, PadChest, Montgomery, and Shenzhen datasets using a different subset from the training set, also achieved an AUC of 0.94. The mean AUC for the Radiological Findings model (RFM), tested on a different subset of the CheXpert training set, was 0.84, and Table 2 displays the AUC and accuracy for each of the nine identified labels, besides the LAM and the TBM results too. Figure 3 shows the traning and validation learning curves for the three models.

**Table 2:** Accuracy and area under the receiver operating characteristic curve (AUC) metrics results for each label of the three models.

**Model**	**Label**	**Accuracy (%)**	**AUC (%)**
LAM	Lung abnormality	89.10 [78.80, 99.30]	93.82 [91.08, 96.56]
TBM	Tuberculosis	88.12 [86.16, 90.16]	94.05 [92.66, 95.44]
RFM	Atelectasis	85.30 [82.29, 88.05]	74.60 [74.15, 74.99]
RFM	Consolidation	89.30 [86.53, 91.59]	79.30 [78.69, 79.84]
RFM	Pleural effusion	86.80 [83.87, 89.38]	91.00 [90.74, 91.21]
RFM	Cardiomegaly	90.10 [87.42, 92.32]	88.20 [87.82, 88.62]
RFM	Edema	85.50 [82.47, 88.19]	84.90 [84.53, 85.20]
RFM	Pneumothorax	85.00 [81.94, 87.75]	86.90 [86.34, 87.43]
RFM	Lung opacity	74.30 [70.62, 77.68]	79.90 [79.64, 80.19]
RFM	Lung lesion	88.60 [85.82, 91.00]	81.90 [81.23, 82.53]
RFM	Mediastinal widening	88.80 [85.99, 91.15]	81.50 [80.95, 82.04]

**Figure 4:**
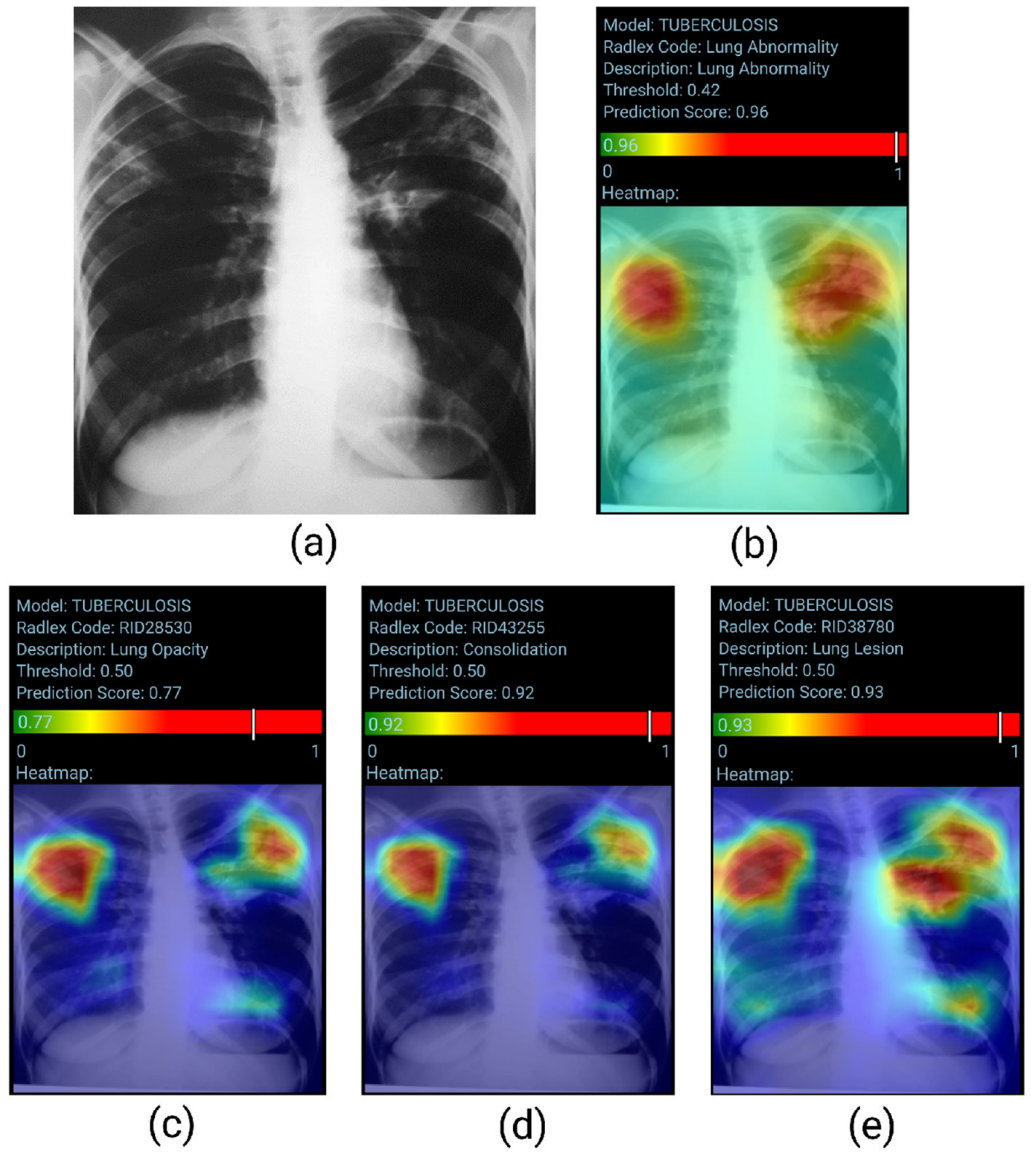
True positive sample for the Lung Abnormality and the Radiological Findings Models. **(A)** Shows original chest-x-ray, **(B)** shows the LAM result, and **(C–E)** show the result for three findings for RFM.

Figure 4 presents a visual representation of the model's performance for Lung Abnormality classification and Radiological Findings classification. The chest radiograph (posteroanterior view) shows heterogeneous opacities/consolidations in both upper lung areas. In addition, a slightly nodular opacity is observed in the left hilum. The LAM returns a high prediction score (0.96), indicating an abnormal radiograph. The RFM correctly classified the abnormalities present in the chest X-ray, with probability scores for opacity, consolidation, and lung lesion respectively of 0.77, 0.92, and 0.93. The heatmap also accurately highlighted the regions of interest.

**Figure 5:**
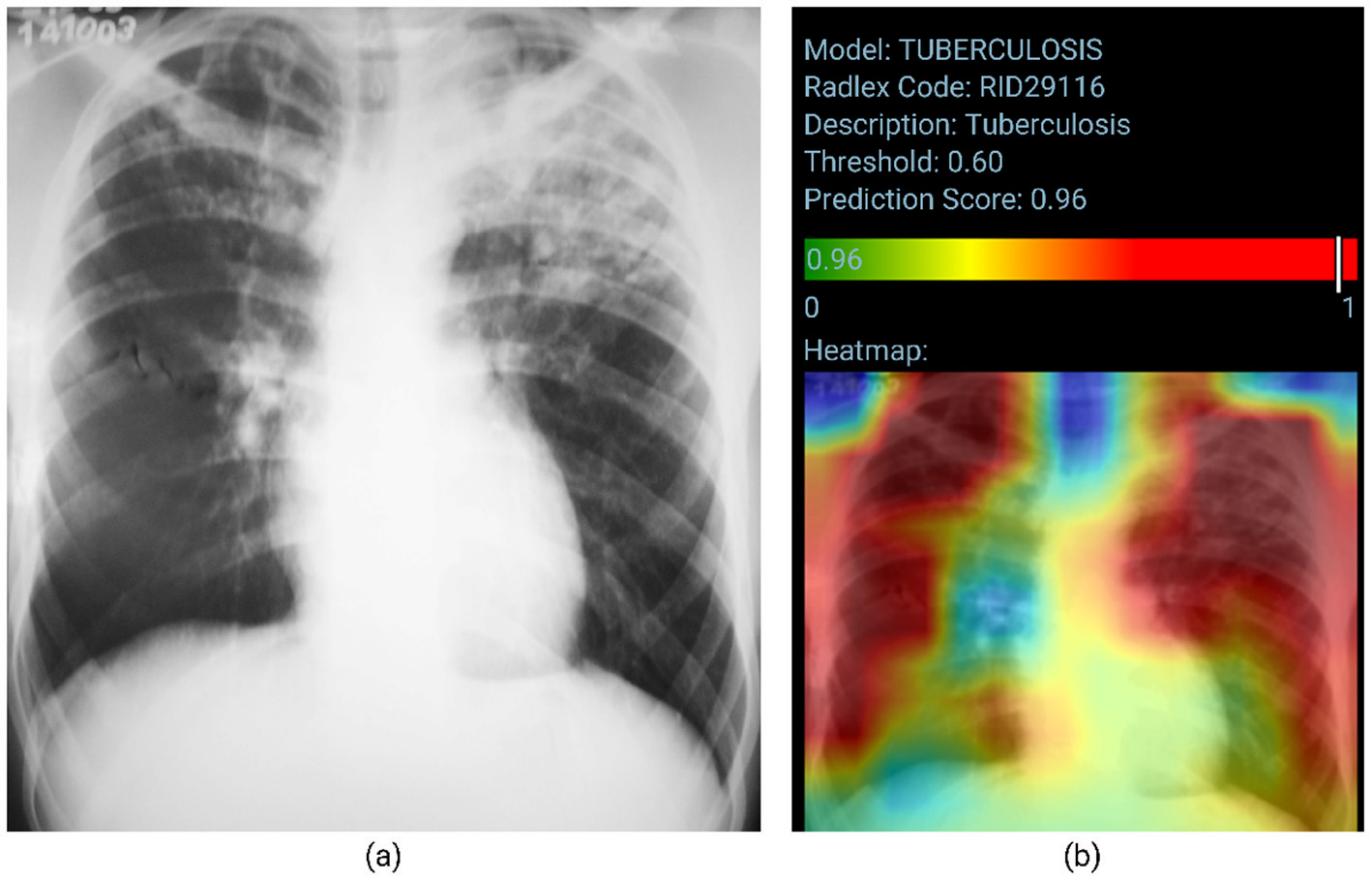
True positive sample for the Tuberculosis Model. **(A)** Shows the original chest x-ray and **(B)** shows the TBM result.

Figure 5 visually represents the model's performance for Tuberculosis classification. Posteroanterior chest radiograph shows heterogeneous consolidative opacities and possible cavities in the bilateral upper lung fields, more extensive and confluent on the left. The radiographic findings were suggestive of an infectious pulmonary process, and a granulomatous etiology was suspected. The patient underwent two sputum smears and culture, with a confirmed diagnosis of pulmonary tuberculosis. The TBM correctly classifies the image (probability score of 0.96). However, the heatmap does not accurately identify the altered areas, as the involvement is predominantly in the upper lung fields.

### 3.2 Validation study results

Group A consisted of two 1st-year radiology residents, three 2nd-year radiology residents, one 3rd-year radiology resident, eight 4th-year radiology residents, one thoracic radiologist (<4 years of experience), four pulmonologists, one infectiologist, one intensivist, two family physicians, two pediatricians, one neurologist, and one general practitioner. Group B consisted of six thoracic radiologists with more than 2 years of experience.

In total, 586 images from 529 patients were completely analyzed by Group A and Group B ( ≤ 2 years of experience). The patient sample had a male-to-female ratio of 222/283, with a mean age of 51.97 years ± 20.03 SD. The number of positive cases for the classes is 164 for consolidation, 136 for Atelectasis, 403 for lung opacity, 298 for lung lesion, 32 for edema, 11 for pneumothorax, 172 for pleural effusion, 80 for cardiomegaly, 31 for mediastinal widening, and 113 for Tuberculosis, and 165 images had no findings.

Tables 3, 4 provide a comprehensive comparison of the results obtained by Group A and the models, using the ground truths determined by Group B. The evaluation metrics used include accuracy, sensitivity, specificity, PPV, and NPV.

**Table 3:** Comparison of accuracy, sensitivity, and specificity metrics between Group A and Algorithm for each label (according to the ground truth of Group B).

**Model**	**Label**	**Accuracy (%)**	**Sensitivity (%)**	**Specificity (%)**
**Lung abnormality**				
LAM	Group A	**88.86 [86.65, 90.83]**	**91.86 [89.58, 93.77]**	79.17 [73.13, 84.38]
	Algorithm	51.20 [47.91, 54.48]	36.29 [32.72, 39.97]	**99.54 [97.45, 99.99]**
**Tuberculosis**				
TBM	Group A	**65.43 [62.20, 68.56]**	68.52 [58.88, 77.12]	**65.00 [61.54, 68.35]**
	Algorithm	51.13 [47.78, 54.46]	**73.15 [63.76, 81.22]**	48.08 [44.52, 51.65]
**Atelectasis**				
RFM	Group A	**82.53 [79.25, 85.48]**	**40.00 [30.98, 49.55]**	92.59 [89.89, 94.76]
	Algorithm	82.20 [78.9, 85.17]	13.04 [7.49, 20.60]	**98.56 [97.05, 99.42]**
**Consolidation**				
RFM	Group A	**84.19 [81.03, 87.02]**	61.90 [51.91, 71.21]	**88.91 [85.81, 91.54]**
	Algorithm	71.21 [67.41, 74.81]	**80.00 [71.07, 87.17]**	69.35 [65.09, 73.39]
**Pleural effusion**				
RFM	Group A	90.68 [88.07, 92.88]	63.87 [54.55, 72.47]	97.30 [95.43, 98.56]
	Algorithm	**91.18 [88.62, 93.32]**	**65.55 [56.28, 74.02]**	**97.51 [95.69, 98.71]**
**Cardiomegaly**				
RFM	Group A	92.85 [90.48, 94.77]	54.69 [41.75, 67.18]	97.39 [95.66, 98.57]
	Algorithm	**94.51 [92.37, 96.19]**	54.69 [41.75, 67.18]	**99.26 [98.10, 99.80]**
**Edema**				
RFM	Group A	94.68 [92.57, 96.33]	34.78 [16.38, 57.27]	97.06 [95.33, 98.28]
	Algorithm	**95.17 [93.14, 96.74]**	**39.13 [19.71, 61.46]**	**97.40 [95.76, 98.54]**
**Pneumothorax**				
RFM	Group A	**98.50 [97.18, 99.31]**	25.00 [3.19, 65.09]	**99.49 [98.53, 99.90]**
	Algorithm	86.02 [82.99, 88.70]	**87.50 [47.35, 99.68]**	86.00 [82.95, 88.70]
**Lung opacity**				
RFM	Group A	73.21 [69.48, 76.71]	**63.42 [57.21, 69.32]**	80.52 [75.94, 84.58]
	Algorithm	**73.88 [70.17, 77.35]**	50.58 [44.3, 56.85]	**91.28 [87.78, 94.04]**
**Lung lesion**				
RFM	Group A	**79.20 [75.73, 82.38]**	67.32 [60.44, 73.69]	**85.35 [81.48, 88.69]**
	Algorithm	70.88 [67.07, 74.49]	**72.20 [65.53, 78.21]**	70.20 [65.43, 74.67]
**Mediastinal widening**				
RFM	Group A	92.51 [90.11, 94.49]	19.35 [7.45, 37.47]	**96.49 [94.63, 97.84]**
	Algorithm	92.51 [90.11, 94.49]	**29.03 [14.22, 48.04]**	95.96 [94.01, 97.43]

The values in brackets represent the 95% confidence interval. Results in bold represent the best performance between physicians and models, considering mean results.

**Table 4:** Comparison of Positive Predictive Value (PPV) and Negative Predictive Value (NPV) between Group A and the Algorithm for each label (according to the ground truth of Group B).

**Model**	**Label**	**PPV (%)**	**NPV (%)**
**Lung abnormality**			
LAM	Group A	94.41 [94.40, 94.43]	**72.53 [72.58, 72.51]**
	Algorithm	**98.93 [98.87, 98.95]**	56.85 [56.90, 56.83]
**Tuberculosis**			
TBM	Group A	21.66 [21.63, 21.69]	72.53 [72.58, 72.51]
	Algorithm	**22.36 [22.33, 22.38]**	**89.56 [89.53, 89.53]**
**Atelectasis**			
RFM	Group A	**35.79 [35.61, 35.88]**	93.74 [93.75, 93.74]
	Algorithm	22.25 [22.13, 22.25]	**97.28 [97.27, 97.29]**
**Consolidation**			
RFM	Group A	**44.65 [44.61, 44.72]**	**94.01 [93.99, 94.02]**
	Algorithm	39.16 [39.12, 39.23]	93.48 [93.45, 93.49]
**Pleural Effusion**			
RFM	Group A	79.03 [78.96, 79.11]	94.28 [94.27, 94.29]
	Algorithm	**81.12 [81.01, 81.22]**	**94.42 [94.40, 94.43]**
**Cardiomegaly**			
RFM	Group A	58.41 [58.31, 58.53]	96.94 [96.93, 96.95]
	Algorithm	**82.82 [82.63, 82.96]**	**97.05 [97.04, 97.06]**
**Edema**			
RFM	Group A	14.44 [14.30, 14.55]	**99.04 [99.04, 99.04]**
	Algorithm	**19.43 [19.34, 19.50]**	99.00 [98.99, 99.00]
**Pneumothorax**			
RFM	Group A	**14.35 [14.32, 14.67]**	99.74 [99.74, 99.74]
	Algorithm	08.07 [08.05, 08.15]	**99.80 [99.79, 99.80]**
**Lung opacity**			
RFM	Group A	65.91 [65.84, 65.96]	79.26 [79.24, 79.29]
	Algorithm	**71.01 [70.93, 71.07]**	**81.98 [81.96, 82.01]**
**Lung lesion**			
RFM	Group A	**64.94 [64.90, 65.00]**	**99.74 [99.74, 99.74]**
	Algorithm	56.49 [56.45, 56.56]	82.86 [82.83, 82.89]
**Mediastinal widening**			
RFM	Group A	05.86 [05.77, 05.92]	**99.09 [99.09, 99.09]**
	Algorithm	**10.90 [10.85, 10.94]**	98.80 [98.80, 98.80]

The values in brackets represent the 95% confidence interval. Results in bold represent the best performance between physicians and models, considering mean results.

Both physicians and the models exhibited statistical differences in terms of accuracy across specific labels: lung abnormality, tuberculosis, consolidation, pneumothorax, and lung lesion (Table 3).

In each case, physicians achieved superior results. Conversely, for the other six findings (atelectasis, pleural effusion, cardiomegaly, edema, lung opacity, and mediastinal widening), statistical equivalence was observed. Concerning sensitivity, differences were observed only for lung abnormality and atelectasis, with physicians achieving higher results. For the remaining nine findings, the results were statistically equivalent. Regarding specificity, statistical differences emerged in seven findings, with physicians outperforming the model in four (tuberculosis, consolidation, pneumothorax, and lung lesion), while the model excelled in three others (lung abnormality, atelectasis, and lung opacity). Results were statistically equivalent to the remaining four findings.

Regarding the PPV metric, Table 4 shows statistical differences arose in ten findings, with physicians achieving higher results in four (tuberculosis, consolidation, pneumothorax, and lung lesion) and the model in the remaining six (lung abnormality, atelectasis, and cardiomegaly). Notably, pleural effusion yielded statistically equivalent results. Lastly, considering the NPV metric, disparities were observed in four findings, with the physicians achieving higher results in three (lung abnormality, atelectasis, and lung opacity) and the model achieving a higher result for consolidation. For the remaining seven findings, the results were statistically the same.

Considering the level of agreement between Group A and Group B with respect to the algorithm, Table 5 presents the agreement with finding's localization and the influence of the model on the physician's interpretation, Group A agreed to a higher degree with the locations represented by the model (37.56%). Group B, on the other hand, showed higher partial agreement with the location of the findings (38.93%).

**Table 5:** Agreement level of physicians in Group A and Group B based on their observations of the AI results.

**Agreement options**	**Group A**	**Group B**
**Agreement with findings' localization**		
Yes	37.56%	21.75%
No	13.19%	14.67%
Partial agreement	24.37%	38.93%
NA	24.87%	24.63%
**Influence of the model on the physician's interpretation**		
No	93.15%	94.54%
Yes (localization)	1.61%	1.31%
Yes (classification)	4.02%	3.13%
Yes (complete)	1.20%	1.00%

Group A exhibited higher agreement with the model in identifying findings in the right lung apex region (13.75%), lower right lung third (11.46%), and middle right lung third (9.77%). In contrast, Group B showed a higher agreement rate in the right lung apex region (14.03%), lower right lung third (11.84%), and right lung apex region (10.72%). These findings indicate variations in the specific areas of agreement between physicians and the model, highlighting differences in their assessments of the precise locations.

## 4 Discussion

In this retrospective study, it was observed that when utilizing the labels generated by Group B, the algorithms performed well for classifying certain findings, while Group A performed well for others, in terms of accuracy, sensitivity, specificity, PPV, and NPV. Upon identifying and analyzing patterns within the retrospective clinical validation process of an artificial intelligence algorithm with Group A in interpreting chest radiographs, it is noted that this study provides a basis to guide future research. However, larger results gap exists, particularly for the Lung Abnormality label. Overall, in terms of metrics, statistically, Group A outperformed the model in more cases – 17 compared to the algorithm's 11 cases (with 26 cases showing statistically equivalent results), Tables 3, 4. Despite the alternation between Group A and AI in the potential to detect some findings, it was noteworthy that both Group A and Group B declared that the algorithm had minimal influence on their opinion in most cases (Table 5).

In terms of comparing the results between the internal and external validation of the models, the Lung Abnormality Model exhibited the most substantial results decline among the three models that constitute the algorithm. As for the Tuberculosis Model, its results decrease from internal to external validation was observed, yet it remains comparable to the results of Group A during clinical validation.

To address the inherent label errors associated with automatically labeled datasets, a rigorous annotation process was implemented, enhancing our external validation methodology by establishing a trustworthy ground truth with the collaboration of the board-certified radiologists (Group B) (Irvin et al., 2019; Bustos et al., 2020; Reis, 2022).

Regarding sensitivity, the models demonstrated equal or higher predictive power than Group A in 8 out of the 11 classes, including tuberculosis, consolidation, and lung lesions. Similarly, in terms of specificity, the models exhibited equal or higher predictive power than Group A in 6 out of the 11 classes, such as cardiomegaly, edema, and lung opacity. Considering the positive predictive values metric, the models were comparable or superior to Group A in 7 out of the 11 classes. Lastly, for the negative predictive values metric, Group A was comparable or superior to the model in 6 out of the 11 classes, including cardiomegaly, pneumothorax, and pleural effusion. In terms of accuracy, the models exhibited equivalent or superior classification capacity compared to Group A in 5 out of the 11 possible classes, as highlighted in Table 3. Notably, this was observed for cardiomegaly, edema, and lung opacity.

Our study revealed low agreement between both physician groups and the models' results. This is particularly evident when evaluating the model for the classification of pulmonary abnormalities, indicating the need for further investigation and potential retraining of the model. Additionally, Group A showed a tendency to agree more with the model's heatmaps compared to the experts, but it is important to acknowledge that the model utilized in this study is a classification model and not a detection model. Therefore, the generated heatmaps should be interpreted as a general indication of the contributing areas for classification rather than precise localization maps for specific abnormalities.

It is worth noting that most physicians agreed that the models did not significantly impact or alter their interpretation of chest radiographs. This suggests that, despite the model's outputs, physicians believe that they maintained independent decision-making processes in diagnosing and interpreting medical images, which does not entirely align with previous literature studies that often report the augmentation of physicians' decision-making skills through AI-supported analysis (Hosny et al., 2022; Ueda et al., 2021; Nam et al., 2019; Sim et al., 2020). However, further research and exploration are necessary to fully understand the potential benefits and limitations of incorporating AI models into clinical practice and to address any concerns or challenges that may arise in the future.

Due to the algorithm's specific focus on supporting general practitioners within the public health system, we emphasized addressing geographic variation and considering local patient characteristics and manifestations. To ensure the algorithm's applicability in this context but also in countries with similar demographics and healthcare systems, we deliberately incorporated a Brazilian dataset [BRAX (Reis, 2022)] during the validation process.

Additionally, as the algorithm was specifically designed to support generalist physicians in their diagnostic process, we assessed agreement among physicians with varying experience levels in interpreting chest radiographs. This analysis aimed to provide valuable insights into the algorithm's results and potential impact on clinical practice across different medical specialties and healthcare scenarios. Therefore, our clinical validation sought to evaluate the agreement between physicians (including board-certified thoracic radiologists, Group B, and Group A) and the algorithm, using the Group B analysis as the ground truth.

There are some limitations to this study. First, external validation posed significant challenges, particularly the complexity of the ground truth generation in this study through the board-certified radiologists. Furthermore, it is important to acknowledge that physicians' perception of AI as a tool for diagnostic imaging may not be universally positive. While the potential benefits of AI in healthcare are highly promising, there exist certain apprehensions that have fostered a cautious stance toward its adoption. These concerns may include issues related to sharing sensitive data, the inherent challenge of elucidating the internal mechanisms of AI models, and apprehensions regarding potential effects on the doctor-patient relationship (Sarwar et al., 2019; Oh et al., 2019; Wadhwa et al., 2020; Huang et al., 2021). Second, the timing of the display of the AI algorithm's results. The AI result is instantly delivered to the reader in the current validation procedure, and despite the team's training, there are no guarantees that the observer will only check this result after his interpretation. This practice can generate biases such as a change of opinion by the physician, persuaded after viewing the AI results (Bernstein et al., 2023). Third, regarding the algorithm's results, the models' classification paradigm poses challenges when attempting to qualitatively generalize the specific patterns underlying the algorithm's errors. This challenge stems primarily from the limitations of using heatmaps, which provide insights into the regions that contributed most to the classification score but lack the comprehensive information offered by detection bounding boxes. Consequently, drawing firm conclusions regarding the reasons behind the algorithm's errors becomes more challenging in comparison to utilizing detection bounding boxes. Fourth, regarding the low number of epochs used during the training and other hyperparameters, they were empirically adjusted to optimize performance and avoid overfitting of models. Fifth, the performance analysis across different physician subgroups–including residents, pulmonologists, infectious disease specialists, and thoracic radiologists–was not comprehensively explored, limiting insights into subgroup-specific variations and their comparative diagnostic accuracy.

Another limitation of this study is the low PPV observed for the Pneumothorax class in the retrospective validation phase. This finding is primarily attributed to the low prevalence of confirmed pneumothorax cases in the retrospective dataset, with only 8 out of 586 cases being true positives. It is well-established that PPV is highly influenced by the prevalence of the target condition in the test population, and low prevalence can lead to disproportionate rates of false positives even in models with high sensitivity and specificity. This limitation is not unique to this study, as previous research has demonstrated similar challenges when evaluating AI models for unusual conditions in general/healthy populations (Ball et al., 2015).

Despite the challenges and limitations discussed, the next steps involve refining the algorithm to achieve more accurate and reliable results. Furthermore, a prospective validation phase is essential, wherein data will be collected in real-time to evaluate the model within the routine of both doctors and patients. By continuously improving the algorithm and conducting prospective validation, we aim to ensure its effectiveness and seamless integration into the regular practice of healthcare professionals. Following this, the next phase will involve clinical trials, during which a fairness analysis will be conducted to evaluate the algorithm's performance across diverse demographic and clinical subgroups, ensuring equitable applicability and effectiveness.

## 5 Conclusion

This retrospective clinical validation study aimed to assess the effectiveness of an AI algorithm in aiding the interpretation of chest X-rays (CXRs) by comparing its performance with physicians of varying levels of experience, using ground truth analyses as references. Although the models used are based on previously established architectures, the innovation of this study lies in the validation methodology that integrates evaluations from physicians of different specialties. By applying the proposed methodology, we compared the interpretations of chest radiographs by physicians in Group A and the predictions of the AI models with the ground truths generated by Group B. This work addresses the gap by involving non-specialist and specialist physicians with varying levels of chest radiograph interpretation expertise in validating an AI algorithm designed to predict pulmonary abnormalities, primarily focusing on tuberculosis and other radiological findings. The presented quantitative and qualitative results support the validation.

The results corroborated this study's hypothesis. The evidence and analyses demonstrate that it is feasible to conduct validation studies involving physicians of different specialties alongside artificial intelligence algorithms for chest X-ray analysis. This confirmation strengthens current understanding and contributes to the existing body of knowledge within the area of clinical validation, particularly in the context of applying AI algorithms.

Both Group A and Group B reported minimal influence of the algorithm on their opinions in most cases. This situation might be a consequence of the need for further refinement of the models, particularly in terms of interpretability. Additionally, some physicians are hesitant to adopt AI because it is a relatively new tool, and they may be reluctant to integrate it into their practice, even though its purpose is to support their decision-making process. Moreover, this study underscores the significance of incorporating real-world data in the clinical validation of AI algorithms, ensuring their robustness and adaptability in diverse healthcare settings. Further investigations are pivotal to establishing the algorithm's viability within the public health system and enhancing its outcomes.

## Data Availability

Publicly available datasets were analyzed in this study. This data can be found here: https://www.nature.com/articles/s41597-022-01608-8. The images usedwere provided from public databases available on the web (BRAX Reis (2022), PadChest Bustos et al. (2020), and Tuberculosis Portals Rosenthal et al. (2017) datasets).
