# Supplementary material for: Clinical validation of an artificial intelligence algorithm for classifying tuberculosis and pulmonary findings in chest radiographs
Source: Front Artif Intell. 2025 Feb 6;8:1512910. doi: 10.3389/frai.2025.1512910 (PMC11843218; doi:10.3389/frai.2025.1512910)
Supplement: Supplementary file 1 [file Data_Sheet_1.pdf]

Supplementary Material

1 SUPPLEMENTARY DATA

Questionnaire used by physicians of Group A and Group B during the chest radiographs evaluation.

1) In your opinion, what is the media type of this X-ray?
( ) Digitized in PACS.
( ) Digital photograph of a film (conventional ”plate” positioned on the lightbox).
( ) Digital photograph of special paper (special glossy paper sheet, not a ”plate”).
( ) Digital photograph of a plain paper sheet
( ) Digital photograph of a monitor.
( ) I don’t know.

2) In your opinion, the technical quality of this radiograph is:
( ) Good.
( ) Fair.
( ) Poor.

3) In your opinion, this chest radiograph is:
( ) Normal
( ) Abnormal

4) If the radiograph is abnormal, mark on the image and in the list below the finding(s) that you believe are present:
[ ] Consolidation
[ ] Pneumothorax
[ ] Atelectasis
[ ] Lung lesion (includes cavities, masses, bronchiectasis, pulmonary nodules—calcified or not, and miliary pattern)
[ ] Lung opacity (includes opacities that cannot be classified as consolidations, e.g., infiltrates)
[ ] Pulmonary edema
[ ] Pleural effusion
[ ] Mediastinal widening
[ ] Cardiomegaly
[ ] Other

5) In your opinion, this radiograph may be:
[ ] Suggestive of pulmonary and/or pleural tuberculosis.
[ ] Suggestive of sequelae of tuberculosis.
[ ] Suggestive of another disease / another radiographic alteration not suggestive of tuberculosis.
[ ] None of the above (normal X-ray).

To proceed with the questionnaire, you should view the algorithm's response. Now, to proceed with the questionnaire, you must view the algorithm's response.

6) After seeing the algorithm's result, do you agree with the classification of normal or abnormal radiograph (look at the ”Pulmonary Abnormality” score—if the score is less than 0.5, then the algorithm considers the exam as normal; if the score is greater than or equal to 0.5, then the algorithm considers the exam as abnormal):
( ) Yes
( ) No

7) If the radiograph is abnormal, do you agree with the location of the finding(s) when viewing the heat map result:
( ) Yes
( ) No
( ) Partially agree
( ) Not applicable (normal exam)

8) If the radiograph is abnormal, do you agree with the heat map result for tuberculosis?
( ) Yes
( ) No
( ) Partially agree
( ) Not applicable (normal exam / Radiograph with pattern not suggestive of TB)

9) If you do not agree or partially agree with the location on the heat map, select the alternative(s) that best represent the location of the finding(s):
[ ] Right lung apex
[ ] Left lung apex
[ ] Right hilum
[ ] Left hilum
[ ] Right costophrenic angle
[ ] Left costophrenic angle
[ ] Middle third of right lung
[ ] Middle third of left lung
[ ] Lower third of right lung
[ ] Lower third of left lung
[ ] Diffuse involvement of right lung
[ ] Diffuse involvement of left lung
[ ] Cardiac area
[ ] Mediastinum

10) Was your personal interpretation influenced by the algorithm's opinion?
( ) No. I maintained my opinion, regardless of the algorithm's opinion.
( ) Yes. I changed my opinion after analyzing the algorithm regarding the location of the finding(s).

( ) Yes. I changed my opinion after analyzing the algorithm regarding the classification of the finding(s).
( ) Yes. I changed my opinion after analyzing the algorithm regarding both the location and classification of the finding(s).

11) What would be your assessment of the difficulty level in interpreting this X-ray?
( ) Easy
( ) Neutral
( ) Difficult
